# Discovery of a Novel *hsp65* Genotype within *Mycobacterium massiliense* Associated with the Rough Colony Morphology

**DOI:** 10.1371/journal.pone.0038420

**Published:** 2012-06-05

**Authors:** Byoung-Jun Kim, Su-Yeon Yi, Tae-Sun Shim, Seung Yeon Do, Hee-Kyung Yu, Young-Gil Park, Yoon-Hoh Kook, Bum-Joon Kim

**Affiliations:** 1 Department of Microbiology and Immunology, Cancer Research Institute, Institute of Endemic Diseases, Seoul National University Medical Research Center (SNUMRC), Seoul National University College of Medicine, Seoul, Republic of Korea; 2 Division of Pulmonary and Critical Care Medicine, Department of Internal Medicine, Asan Medical Center, College of Medicine, University of Ulsan, Seoul, Republic of Korea; 3 Korean Institute of Tuberculosis, Korean National Tuberculosis Association, Chungcheongbuk-Do, Republic of Korea; St. Petersburg Pasteur Institute, Russian Federation

## Abstract

So far, genetic diversity among strains within *Mycobacterium massiliense* has rarely been studied. To investigate the genetic diversity among *M. massiliense,* we conducted phylogenetic analysis based on *hsp65* (603-bp) and *rpoB* (711-bp) sequences from 65 *M. massiliense* Korean isolates. We found that *hsp65* sequence analysis could clearly differentiate them into two distinct genotypes, Type I and Type II, which were isolated from 35 (53.8%) and 30 patients (46.2%), respectively. The *rpoB* sequence analysis revealed a total of four genotypes (R-I to R-IV) within *M. massiliense* strains, three of which (R-I, R-II and R-III) correlated with *hsp65* Type I, and other (R-IV), which correlated with Type II. Interestingly, genotyping by the *hsp65* method agreed well with colony morphology. Despite some exceptions, Type I and II correlated with smooth and rough colonies, respectively. Also, both types were completely different from one another in terms of MALDI-TOF mass spectrometry profiles of whole lipid. In addition, we developed PCR-restriction analysis (PRA) based on the *Hinf* I digestion of 644-bp *hsp65* PCR amplicons, which enables the two genotypes within *M. massiliense* to be easily and reliably separated. In conclusion, two distinct *hsp65* genotypes exist within *M. massiliense* strains, which differ from one another in terms of both morphology and lipid profile. Furthermore, our data indicates that Type II is a novel *M. massiliense* genotype being herein presented for the first time. The disparity in clinical traits between these two *hsp65* genotypes needs to be exploited in the future study.

## Introduction

Rapidly growing mycobacteria (RGM) are ubiquitous organisms increasingly emerging as important human pathogens. Recently, there have been more frequent reports of RGM infections in immunocompetent people as well as in people with predisposing factors or those who are immunosuppressed [1], [2]. In particular, among RGMs, *Mycobacterium abscessus* is commonly associated with wound infection and abscess formation and is the RGM that most frequently causes chronic lung disease. *M. abscessus* is also notable for its resistance to treatment and the poor clinical outcome of infection with the organism [2]. In South Korea, in contrast to countries such as the United States and Japan [3]–[5], infection of *M. abscessus* is the most prevalent RGM infection, and second only to the *M. avium* complex for nontuberculous mycobacterium (NTM) [6], [7].

Recent application of multilocus sequencing has broadened our knowledge about the diversity between the *M. abscessus* complex. Two new species of mycobacteria closely related to *M. abscessus, M. massiliense* and *M. bolletii*, have been described [8], [9]. A recent molecular epidemiologic study using 144 RGM isolates from Korean patients showed that two *M. abscessus* related species, *M. abscessus* and *M. massiliense,* were responsible for the most of the infections [*M. abscessus* (65/144 isolates, 51.2%) and *M. massiliense* (59/144 patients, 46.5%)]. However, *M. bolletii* has rarely been isolated from Korean patients (2/144 patients, 1.6%) [10]. It should be noted that the disparity between *M. abscessus* and *M. massiliense* infections in terms of clinical significance was reported by a recent paper using Korean patients infected with either *M. abscesus* or *M. massiliense*, thereby putting the stress on the separation between the two related species via molecular - based methods in the clinical aspects [11].

As alternatives to 16 S rRNA gene sequencing, several other gene targets have been effectively used for the NTM differentiation, demonstrating the limited value in the separation between some closely related NTM strains [12]–[16]. Among these gene targets, partial sequencing that targeted the *rpoB* or *hsp65* has increasingly been used to differentiate between *M. abscessus, M. massiliense, and M. bolletii* [8], [10], [11], [17], [18].

Due to the lack of clinical or epidemiological information regarding *M. massiliense*, studies about genetic or phenotypic traits of *M. massiliense* have rarely, if ever, been introduced, as compared to *M. abscessus*. Thus, the present study aims to elucidate the genetic diversity between *M. massiliense* clinical isolates by two different chronometer molecules - *hsp65* and *rpoB* - genes and to identify the relationships between the determined genotype and phenetic traits, particularly in terms of colony morphology.

## Results

### Identification by *hsp65* Sequencing Analysis

**Figure 1:**
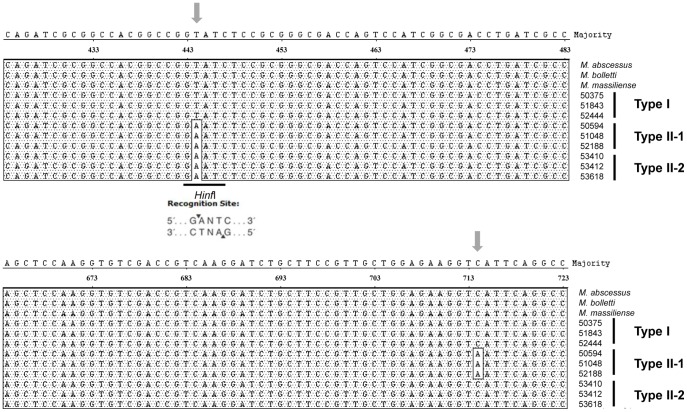
Sequence polymorphisms between the three *hsp6*5 sequevars of *M. massiliense*. Type I strains have the same sequence as the *M. massiliense* type strain; however, Type II-1 and Type II-2 strains differed from the *M. massiliense* type strain by 2-bp (T444A and C714A) and 1-bp (T444A), respectively. The nucleotide numbers correspond to those from the complete sequence of the heat shock protein 65 kDa (*hsp65*) gene of *M. abscessuss* ATCC 19977 (GenBank no. CU458896).

**Figure 2:**
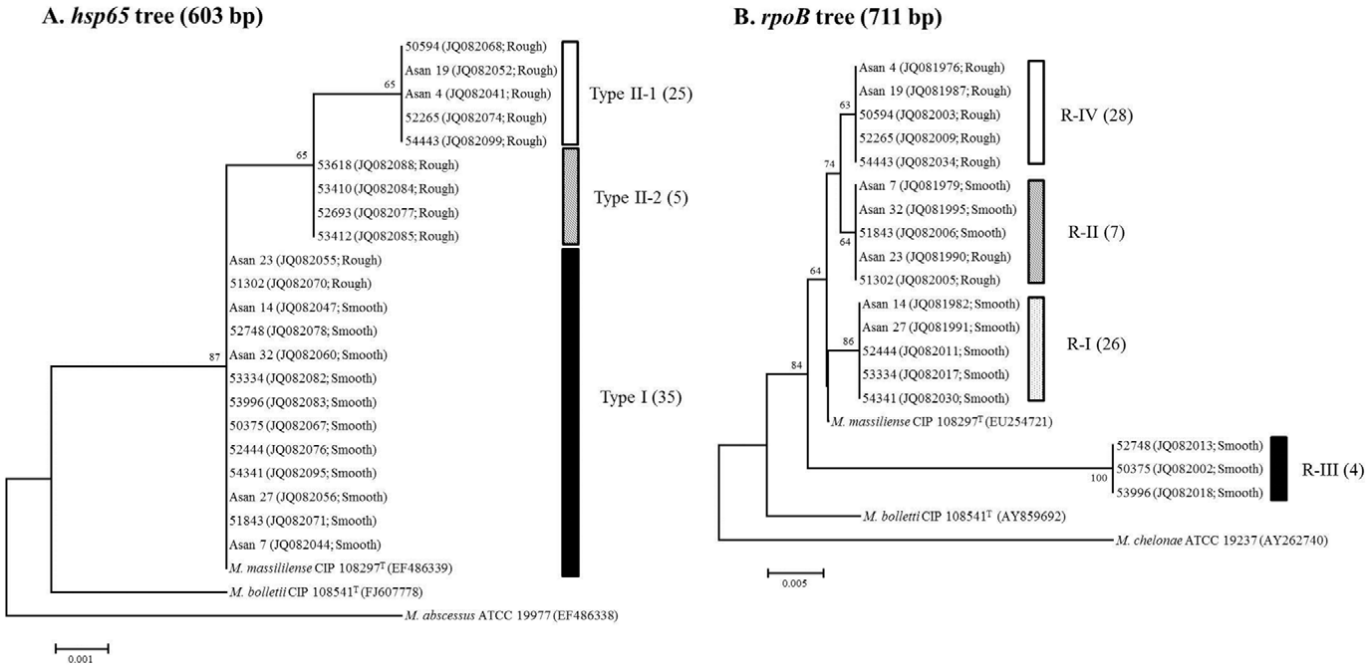
Phylogenetic trees based on the *hsp65* gene (603 bp) and *rpoB* gene (711 bp) sequences. Phylogenetic trees based on the (A) *hsp65* gene (603 bp) and (B) *rpoB* gene (711 bp) sequences from *M. massiliense* clinical isolates, *M. massiliense* CIP 108297^T^, *M. bolletii* CIP 108541^T^, *M. chelonae* ATCC 19237, and *M. abscessus* ATCC 19977. These trees were constructed using the neighbor-joining method. The bootstrap values were calculated from 1,000 replications. Bootstrap values of <50% are not shown. The bars indicate numbers of substitutions per nucleotide position.

First, 109 *M. abscessus* complex strains from Asan medical center (AMC), which had been identified by *rpoB* PRA, were further analyzed using the *hsp65* sequencing method, showing the prevalence of 44 *M. abscessus* (40.4%) and 65 *M. massiliense* strains (59.6%) (Table 1). However, no *M. bolletii* strains were found in our cohort (0%). Phylogenetic analysis based on the partial *hsp65* sequence (603 bp) showed that there were two phylogenetic groups (Type I and II) within the 65 *M. massiliense* strains, composed of three sequevars (Figure 1 and 2A). Type I isolated from 35 patients (53.8%) included a single sequevar having the same 603-bp *hsp65* sequences as *M. massiliense* CIP 108297^T^. But Type II, also isolated from 30 patients (46.2%), included two different sequevars, Type II-1 and Type II-2, which showed 2-bp (T444A and C714A) and 1-bp different sequences (T444A) from *M. massiliense* CIP 108297^T^, respectively (Table 2, Figure 1). Type I, Type II-1, and Type II-2 were isolated from 35 (53.8%), 25 (38.5%) and 5 patients (7.7%), respectively (Figure 2A).

**Table 1:** Separation of 109 *M. abscessus* related Korean strains used in this study into species or genotype level by sequence analysis based on the partial *hsp65* gene sequence (603 bp).

Species and group	No. (%) of strains
***M. abscessus***	**44 (40.4)**
***M. massiliense***	**65 (59.6)**
**Type I**	**35 (32.1)**
**Type II**	**30 (27.5)**
**Total**	**109 (100.0)**

**Table 2:** The frequency of the two *hsp65* genotypes (Type I and Type II) determined by *hsp65* sequence analysis and *Hinf* I PRA methods, and the two colony morphotypes (rough and smooth) among 65 *M. massiliense* clinical strains.

		*Hinf* I PRA		Colony morphotype	
*hsp65* genotype^a^	No. (%)^b^	366, 278	280, 86, 278	Rough	Smooth
**Type I**	**35 (30.1)**	**35 (100.0)**	**0 (0.0)**	**12 (34.3)**	**23 (65.7)**
**Type II**	**30 (27.4)**	**0 (0.0)**	**30 (100.0)**	**30 (100.0)**	**0 (0.0)**

a The *hsp65* genotypes were determined by *hsp65* sequence analysis (603-bp).

b The percentage was calculated among *M. massiliense* strains.

### *rpoB* Sequence Analysis of *M. massiliense* Strains

The partial *rpoB* sequences (711-bp) from the 65 *M. massiliense* strains were compared with each other to check the genetic heterogeneity between them. The *rpoB* based sequence analysis revealed the presence of four groups within the 65 *M. massiliense* strains, suggesting more genetic diversity within *M. massiliense* strains in the 711-bp *rpoB* sequence than in the 603-bp *hsp65* sequence. All of the isolates showed different *rpoB* sequences than the *M. massiliense* CIP 108297^T^ with sequence divergence ranging from 2-bp (R-I, R-II, and R-IV) to 14-bp (R-III). These groups were clearly separated by phylogenetic analysis based on *rpoB* gene sequences of the 65 *M. massiliense* strains (Figure 2B). Despite some minor exceptions, the genotype R-I, R-II, R-III and R-IV of four *rpoB* genotypes were related to the *hsp65* Type I and Type II, respectively. The R-I were found most frequently in *M. massiliense* Type I (68.6%, 24/35 strains). The R-IV, which had an *rpoB* sequence that was 2-bp different *rpoB* sequence (T2760C and G2907A) from *M. massiliense* CIP 108297^T^ were most frequently found in *M. massiliense* Type II (93.3%, 28/30 strains), but not in Type I (Table 3).

**Table 3:** The frequency of the four *rpoB* genotypes (R-I to R-IV) determined by *rpoB* sequence analysis (711-bp) among 65 *M. massiliense* clinical strains, polymorphisms of *rpoB* genotypes and relationships between *rpoB* and *hsp65* genotypes.

	No. (%)		
rpoB genotype^a^	*hsp65* Type I	*hsp65* Type II	*P* - value
R-I **(T2484G and G2934A)**	24 (68.6)	2 (6.7)	0.010
R-II **(C2569T and T2851C)**	7 (20.0)	0 (0.0)	0.000
R-III **(C2475T, T2484C, T2835G, C2848G,** **A2849C, G2850C, C2853T, C2859T, A2861C,** **G2862A, G2868C, C2869A, A2870C,** **G2871C, G2874T, T2877G, C2880G,** **C2886T, C2988T, and C3022G)**	4 (11.4)	0 (0.0)	0.056
R-IV **(T2760C and G2907A)**	0 (0.0)	28 (93.3)	0.000
Total	35 (100.0)	30 (100.0)	

a Polymorphisms which are different from the type strain of *M. massiliense*. The nucleotide numbers correspond to those from complete sequence of RNA polymerase beta subunit (*rpoB*) gene of *M. abscessus* ATCC 19977 (GenBank no. CU458896).

### Relationships between *hsp65* Genotypes and Colony Morphology

**Figure 3:**
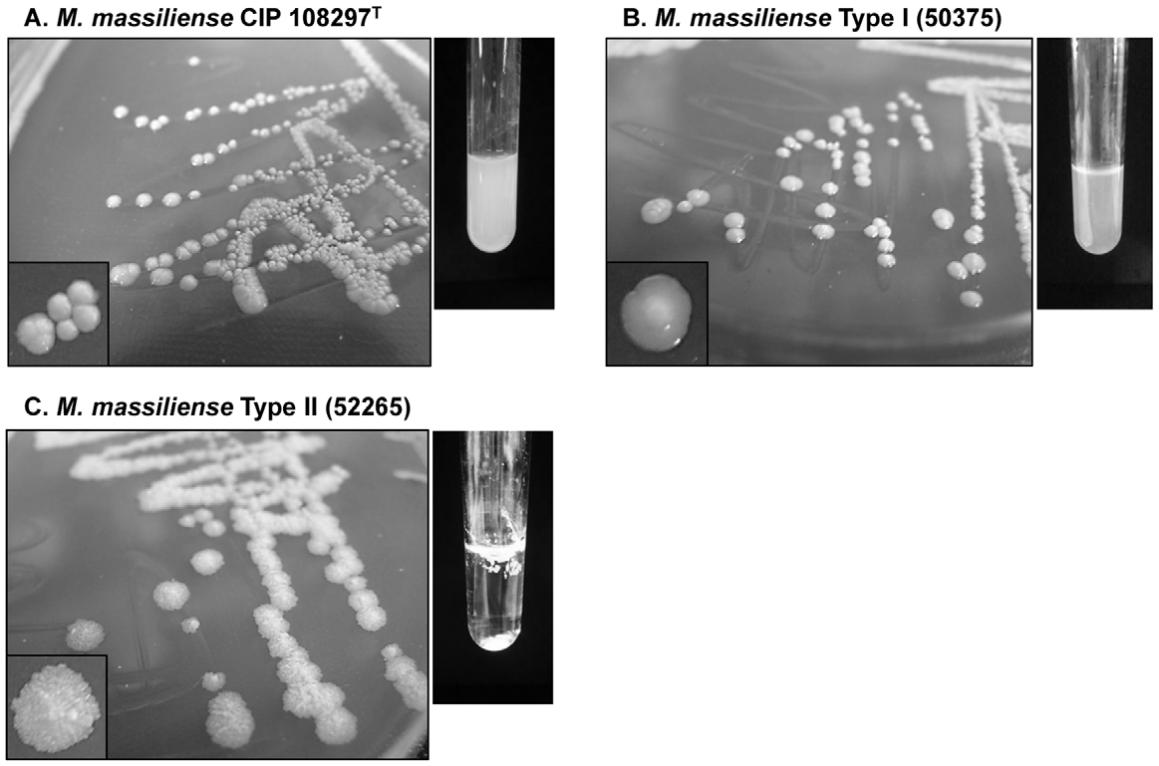
Colony morphology and the growth patterns in 7H9 broth medium. Colony morphology (left panel) and the growth patterns in 7H9 broth medium (right panel) of (A) *M. massiliense* CIP 108297^T^, (B) *M. massiliense* Type I strain, and (C) *M. massiliense* Type II strain.

Sub-cultured colonies of the 65 *M. massiliense* strains identified by *hsp65* sequence analysis and *M. massiliense* CIP 108297^T^ were analyzed on 7H10 agar plates. Notably, substantial differences in colony morphology were found between two *hsp65* genotypes, Type I and Type II (Figure 3). Interestingly, all the 30 of the isolates belonging to Type II (Type II-1 and Type II-2) showed rough colony without any exception. However, the Type I isolates showed both rough and smooth colony morphologies. In Type I, as shown in *M. massiliense* CIP 108297^T^, smooth morphology was more common than rough morphology [smooth vs. rough; 23/35 isolates (65.7%) vs. 12/35 isolates (34.3%)] (Table 2). In addition to the different colony morphologies in the agar plates, Type I of smooth morphotype and Type II of rough morphotype also differed in terms of the growth characteristics of 7H9 broth cultures. While Type I strain and *M. massiliense* CIP 108297^T^ with smooth morphotype showed a dispersed growth pattern, the Type II isolate showed a typical aggregative pellicle growth that is confined to the surface of the medium, as shown in the *M. tuberculosis* clinical isolates (Figure 3). But, the Type I strain with rough morphotype was quite similar to Type II in terms of the growth pattern of the broth culture (data not shown).

### Differentiation between Two Genotypes of *M. massiliense* Strains by *Hinf* I PRA

**Figure 4:**
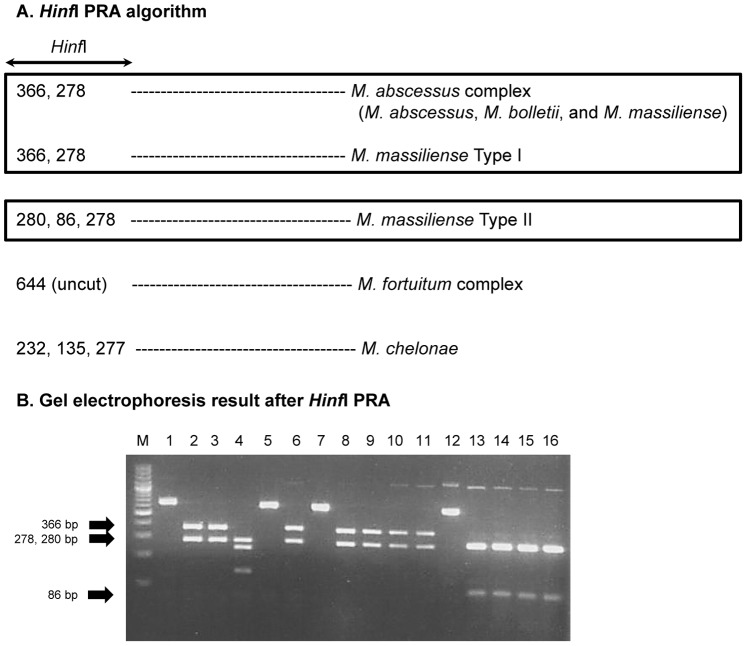
Identification of *M. massiliense* Type I and Type II strains by *hsp65* PRA method. (A) *Hinf* I PRA algorithm for differentiating of *M. massiliense* Type I and Type II strains. (B) Agarose gel electrophoresis after *Hinf* I PRA. Lanes: M, 100-bp ladder; 1, *M. abscessus* (uncut); 2, *M. abscessus* (*Hinf* I cut); 3, *M. bolletii* (*Hinf* I cut); 4, *M. chelonae* (*Hinf* I cut); 5, *M. fortuitum* (*Hinf* I cut); 6, *M. massiliense* (*Hinf* I cut); 7, 50375 (Type I, uncut); 8, 50375 (Type I, *Hinf* I cut); 9, 51302 (Type I, *Hinf* I cut); 10, 51843 (Type I, *Hinf* I cut); 11, 52352 (Type I, *Hinf* I cut); 12, 51048 (Type II, uncut); 13, 51048 (Type II, *Hinf* I cut); 14, 52008 (Type II, *Hinf* I cut); 15, 50594 (Type II, *Hinf* I cut); 16, 52012 (Type II, *Hinf* I cut).

To enable the simple separation of *M. massiliense* Type II strains from other related RGMs, we developed a novel *Hinf* I PRA algorithm. The results and the algorithm obtained by applying the *Hinf* I PRA method to the 65 *M. massiliense*-related strains and five reference strains are summarized in Table 2 and Figure 4A, respectively. As predicted, the *M. massiliense* Type II strains were clearly distinguished from other related RGMs, including *M. massiliense* Type I strains, producing distinct PRA patterns (280, 278, and 86-bp), although it was not possible to separate between the upper two bands, 280 and 278-bp (Figure 4B). When the results of both the *Hinf* I PRA and *hsp65* sequencing methods were compared, the sensitivity and specificity of our *Hinf* I PRA method for separating between the two genotypes of *M. massiliense* were 100% and 100%, respectively (Table 2). The informations about *rpoB* and *hsp65* genotypes, *Hinf* I PRA patterns and colony morphology of each isolate were shown in Table S1.

### Biochemical Tests and Drug Susceptibility Tests

The phenotypic characteristics of three reference strains, four *M. massiliense* Type I strains, and four *M. massiliense* Type II strains were analyzed and compared. With the exception of the colony morphology, no characteristic traits were not found to differentiate between Type I and Type II groups (Table S2). The results of our drug susceptibility test likewise showed no significant differences between Type I and Type II groups (Table S3). In addition, no point mutations were found at the adenine at position 2058 (A2058) or 2059 (A2059) in the peptidyltransferase region of the 23 S rRNA gene in any of the 65 *M. massiliense* strains (data not shown).

### Comparison of HPLC and MALDI-TOF Mass Spectrometry Profiles between Two Genotypes of *M. massiliense*

**Figure 5:**
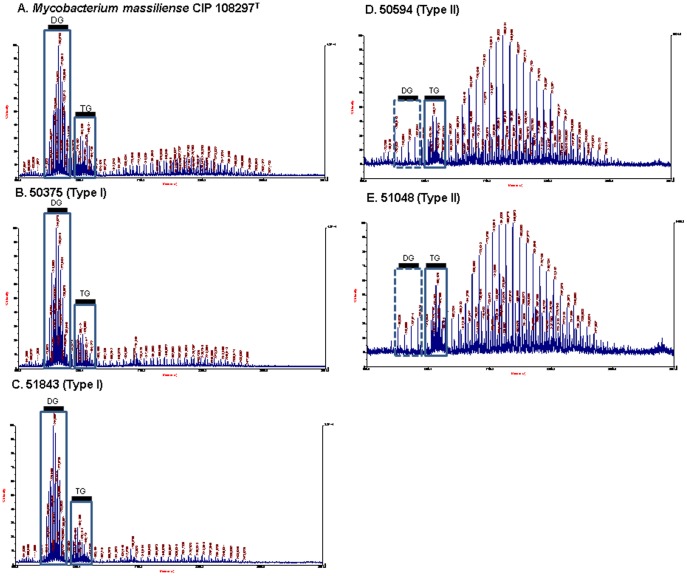
MALDI-TOF mass spectrometry analysis. MALDI-TOF mass spectrometry analysis of extracted lipids from (A) *M. massiliense* CIP 108297^T^, (B) 50375 (Type I), (C) 51843 (Type I), (D) 50594 (Type II), and (E) 51048 (Type II). DG, diglycosylated GPLs; TG, triglycosylated GPLs.

The profiles of HPLC and MALDI-TOF mass spectrometry of *M. massiliense* CIP 108297^T^, four *M. massiliense* Type I strains, and four *M. massiliense* Type II strains were analyzed and compared. Generally, the HPLC profiles of all the *M. massiliense* strains were virtually identical. However, both genotypes showed different profiles at the peak of about 1.91 retention time to be separated with each other. All four of the Type II strains showed a higher level of intensity at this peak than four Type I and *M. massiliense* CIP 108297^T^ (Figure S1). The most substantial differences between the MALDI-TOF mass spectrometry profiles were found between the two genotypes. Usually, the MALDI-TOF mass spectrometry profiles have two distinct clusters of peaks ranging from m/z 1171 to m/z 1316 and from m/z 1360 to m/z 1464. The first cluster and the second cluster represent diglycosylated glycopeptidolipid (GPL) and triglycosylated GPL, respectively [19]. All four of the Type I strains and *M. massiliense* CIP 108297^T^ showed the typical MALDI-TOF mass spectrometry profiles of two clusters, but all four of the Type II strains showed unusual profiles, with a significantly low intensity of the putative diglycosylated GPLs and diverse peaks of high intensity from m/z 1598 to m/z 2477, suggesting a disparity in the GPL nature between the two genotypes (Figure 5).

## Discussion

Following the recent taxonomic separation of three very closely related RGMs - *M. abscessus*, *M. massiliense* and *M. bolletii* - reports regarding human infections of *M. massiliense* have been increasing [20]–[23]. Thus, it has become more important to study the diversity between their interspecies or intraspecies [10], [24]. A recent report based on multilocus sequencing showed that *M. massiliense* is composed of strains with more diverse genetic heterogeneity than its closely related species, *M. abscessus* [24]. To investigate the intraspecies genetic diversity within *M. massiliense*, we studied 65 Korean strains by applying a gene-based sequencing approach consisting of two independent chronometers (*hsp65* and *rpoB* gene).

Rather than 16 S rRNA gene-based analysis, which has been shown to have limited effectiveness in discriminating between mycobacterial strains, a 603-bp *hsp65* sequencing analysis has been proven to be useful for identifying mycobacteria [25]–[27], particularly for the separating between the three related species of *M. abscessus*; *M. abscessus*, *M. massiliense*, and *M. bolletii* [11], [28]. Our *hsp65*-based sequence analysis showed that *M. massiliense* is more prevalent than *M. abscessus* (59.6% vs. 40.4%), supporting the previous report that, in South Korea, *M. massiliense* isolated with a relative higher frequency than in other areas [10]. So, this epidemiologic feature raised the possibility of the presence of a distinct *M. massiliense* strain in South Korea. Indeed, for the first time, we report the identification of a novel *hsp65* genotype (Type II) that was found with a high frequency in our Korean *M. massiliense* isolates (46.2%, 30/65 patients). Since none of the *hsp65* sequences in the NCBI databases perfectly match those of Type II, it seems that Type II may very well be a unique *M. massiliense* strain in Korea.

To investigate the phenotypic difference between two *hsp65* genotypes, a number of phenetic traits were compared between both genotypes, including colony morphology. Although most traits could not provide any definitive criteria for differentiation, a strong correlation was discovered between the colony morphology of the two *hsp65* genotypes. Specifically, while the majority of the *hsp65* Type I genotype (65.7%, 23/35 strains) showed a smooth morphotype, all the 30 strains with *hsp65* Type II showed a rough colony morphology. So, it is tempting to speculate that the rough morphotype of Type II, like *M. tuberculosis*, may be an innate trait that evolved from the smooth strain, rather than a trait acquired via induced mutations during the *in vivo* infection.

NTMs have long been recognized as having both rough and smooth colony phenotypes [29]. Several reports have found a correlation between colony morphology and virulence, with rough variants generally being more virulent than smooth variants [30], [31]. Particularly, in the *M. abscessus* strains, smooth morphotype has occasionally spontaneously reverted to rough morphotype after several passages on agar plates or via *in vivo* passage into mice due to the reduced expression of glycopeptidolipid (GPL) [30]. Although the exact mechanism still remains a mystery, the phenomena may be attributed to the loss of GPL resulting in excessive secretion of TNF-á from the macrophage [32]. Our MALDI-TOF MS analysis showed that the Type I strains with smooth morphotype and Type II strains had completely different lipid profiles. In particular, the peaks corresponding to putative diglycosylated GPL were reduced in the Type II strains as compared to Type I. Given that mycobacterial cell wall lipid is one of the most important factors determining virulence, leading to the modulation of the host immune response, it is likely that two *hsp65* genotypes of *M. massiliense* would show different pathogenesis. However, the above hypothesis needs to be proven through detailed analysis of clinical data and *in vivo* and *in vitro* virulence studies in the future. In addition, we could not find any phenotypic or chemotaxonomic differences between the 11 strains showing *hsp65* Type I genotype with rough phenotype and the 30 Type II strains. The question of whether the two genotypes with rough phenotype (Type I and II) acquire the rough phenotype in different ways should be addressed in a future study.

A previous report showed that sequence analysis based on the 711-bp *rpoB* gene was better than *hsp65* sequence based analysis in terms of discriminating between closely related mycobacterial strains, particularly, strains within *M. abscessus* or *M. massiliense* [12]. Our *rpoB* sequence analysis was also able to separate all 65 of the *M. massiliense* strains into four more diverse genotypes (R-I to R-IV) than *hsp65* sequence analysis. However, while the previous report [10] had discordant results between *hsp65* and *rpoB*-based sequence analysis in species determination in some *M. massiliense* strains, all 65 of the isolates identified as *M. massiliense* by the *hsp65* method were also identified as *M. massiliense* by *rpoB* methods (Table 3, Figure 2B). Phylogenetic analysis based on the *rpoB* sequences showed that most *hsp65* Type II strains also formed a monophyletic clade in a *rpoB* tree (Figure 2B), suggesting that *hsp65* Type II may be composed of genetically identical strains. However, *hsp65* Type I strains were divided into the three different clades in a *rpoB* tree, suggesting that *hsp65* Type I may be composed of more diverse genetic groups. These results provide a likely explanation for the presence of phenotype divergence (the coexistence of both morphotypes) in type I over type II strains.

Although PRA is a previous century technique, replaced by sequencing analysis producing more accurate results, the PRA method has still been used for routine screening purposes in clinical settings for differentiating the mycobacterial strains, due to the its ease and rapidity [33]–[35]. So, we developed a novel PRA method using *Hinf* I enzyme targeting a 644-bp *hsp65* genes for selective identification of *M. massiliense* Type II. A blind test for evaluating this PRA method proved that it can successfully identify all Type II strains from Type I, suggesting its feasibility for the diagnostic or epidemiologic purposes.

Our *hsp65* and *rpoB* based methods failed to differentiate rough and smooth strains within *hsp65* Type I. To overcome this limitation, methods with more discriminatory power such as variable-number tandem repeat (VNTR) [36]–[38] or whole genome sequencing [39]–[41] should be applied into *hsp65* Type I strains for the future study.

In summary, through *hsp65* sequencing analysis of 65 Korean *M. massiliense* strains, we found two *hsp65* genotypes, Type I from 35 patients (53.8%) and Type II from 30 patients (46.2%), which were related to smooth and the rough colony phenotypes, respectively. Furthermore, the two *hsp65* genotypes were also completely different in terms of their lipid profiles by MALDI-TOF MS. A total of four genotypes (R-I to R-IV) were also found by *rpoB* sequencing analysis, three of which (R-I, R-II, and R-III) were related to *hsp65* Type I and the other (R-IV) which was related to *hsp65* Type II. Our data indicates that the Type II *hsp65* genotype, which also shows the R-IV *rpoB* genotype, is a novel *M. massiliense* group introduced for the first time in this study. In addition we developed a novel PRA method for selectively separating *hsp65* Type II from other RGMs. Our data suggests that *M. massiliense* strains may be composed of genetically distinct diverse groups, of which pathogenic potentials need to be evaluated for future study.

## Materials and Methods

### Bacterial Strains

A total of 109 strains from sputa samples of different patients were provided by Tae Sun Shim, Asan Medical Center, South Korea were collected from January 2004 to June 2011. All the experiments were performed just on the extracted DNA the isolated strains, not directly sputa DNA. Furthermore, all the samples were collected in an anonymized manner, and any information about the personal details of the patients and any details about their clinical history were not supplied. In this case, the study could be under the waiver of informed consent. With the documentation for waiver of informed consent, this work was approved by the institutional review board of Seoul National University Hospital (C-1202-057-398) and Asan Medical Center (2012-0170). These samples were proved to be *M. abscessus* complex strains by *rpoB* PRA method [34]. For the further separation at the species level, these isolates were used for re-identification by direct sequencing protocol targeting the partial *hsp65* gene [14]. The type strains of *M. abscessus* (ATCC 19977), *M. massiliense* (CIP 108297), and *M. bolletii* (CIP 108541) were used for comparison.

### Colony Morphology and Broth Culture

To observe the colony morphology, the isolates cultivated on Ogawa media were sub-cultured on 7H10 agar medium supplemented by OADC at 37°C for 3 to 5 days [42]. To examine growth patterns on the broth culture, the clinical isolates and *M. massiliense* type strain were inoculated into 7H9 broth supplemented with ADC at approximately 1×10^4^ CFU/ml and cultured at 37°C for 5 to 7 days [30], [42].

### Biochemical Tests and Drug Susceptibility Tests

To determine their taxonomic relationships, *M. massiliense* CIP 108297^T^, four *M. massiliense* Type I strains (50375, 51843, 52352, and 52444), and four *M. massililense* Type II strains (50594, 51048, 52188, and 52265) were tested for biochemical and drug susceptibility profiles. These strains were cultured into Middlebrook 7H9 broth supplemented by ADC at 37°C. Colony morphology, pigment production in the dark condition, photo-induction, and the ability to grow at various temperatures (25, 37, and 45°C) were analyzed during six-week incubation on Middlebrook 7H10 agar plates. Acid-alcohol fastness was determined by Ziehl-Neelsen and auramine O staining. Other tests measured: niacin accumulation, nitrate reductase, arylsulfatase on days 3 and 14, heat-stable catalase (pH 7, 68°C), tellurite reductase, Tween 80 hydrolysis, urease and pyrazinamidase (PZA) [43]. Additional biochemical tests, such as to detect the activity of alkaline phosphatase, esterase (C4), esterase lipase (C8), lipase (C14), leucine arylamidase, valine arylamidase, and crystine arylamidase were conducted with the API ZYM kit (bioMerieux) as recommended by the manufacturer.

Inhibition tests including tolerance to thiophene-2-carboxylic acid hydrazide (TCH), p-nitrobenzoate (PNB), 5% sodium chloride, ethambutol (EMB) and picric acid were carried out, and the ability to grow on MacConkey agar without crystal violet was examined. Also, antimicrobial susceptibility was determined by the agar proportion method on 7H10 medium [43].

### High-performance Liquid Chromatography (HPLC) Analysis

HPLC was used to analyze mycolic acids from *M. massiliense* CIP 108297^T^, the four aforementioned *M. massiliense* Type I strains, and the four aforementioned *M. massililense* Type II strains as previously described [44]. The Microbial Identification system (MIDI Inc.) and the HPLC mycobacterium library (available online at http://www.MycobacToscana.it) were used to identify mycolic acid patterns.

### Matrix-Assisted Laser Desorption Ionization-Time of Flight (MALDI-TOF) Mass Spectrometry Analysis

To analysis MALDI-TOF mass spectrometry, lipids were extracted with CHCl_3_/CH_3_OH (1∶1 v/v, adding 0.5 µl of 2,5-dihydroxybenzoic acid) from 30 ml 7H9 broth cultures of *M. massiliense* CIP 108297^T^, the four *M. massiliense* Type I strains, and the four *M. massililense* Type II strains. MALDI-TOF mass spectrometry was performed on the extracted samples with a Voyager DE-STR MALDI-TOF instrument (Perseptive Biosystems) equipped with a pulse nitrogen laser emitting at 337 nm as previously described [45].

### DNA Extraction and PCR

Total DNAs were extracted from cultured colonies using the bead beater-phenol extraction method [14], and then used as templates for PCR. To study genetic variations of the *M. massiliense* related strains, two independent target genes - *hsp65* and *rpoB* - were amplified. To amplify partial gene (644 bp), the *hsp65* PCRs were applied to a total of 109 clinical isolates. A set of primers HspF3 (forward; 5′-ATC GCC AAG GAG ATC GAG CT-3′) and HspR4 (reverse; 5′-AAG GTG CCG CGG ATC TTG TT-3′) was used [14]. To amplify partial gene (1092 bp), the *rpoB* PCRs (with a slight modification of previous methods) [12], were applied to 65 clinical isolates, which had already been identified into *M. massiliense* strains by the *hsp65* sequencing protocol. Primer sets of MycoF2 (forward; 5′-ATC GCC GAC GGT CCC TGC-3′) and MycoR2 (reverse; 5′-GAA CCG CTG GCC ACC GAA CT-3′) were used for PCR. The template DNA (50 ng) and 20 pmol of each primer were added to a PCR mixture tube (AccuPower PCR PreMix; Bioneer, Daejeon, South Korea) containing one unit of Taq DNA polymerase, 250 µM of deoxynucleotide triphosphate, 10 mM Tris-HCl (pH 8.3), 10 mM KCl, 1.5 mM MgCl_2_, and gel loading dye. The final volume was adjusted to 20 µl with distilled water, and the reaction mixture was then amplified as previously described [14], [46] using a model 9700 Thermocycler (Perkin-Elmer Cetus).

### *Hinf* I PCR Restriction Fragment Length Polymorphism Analysis (PRA) Targeting 644 bp *hsp65* gene

A PRA algorithm using the *Hinf* I enzyme to differentiate *M. massiliense* Type II from *M. massiliense* Type I, as well as other *M. massiliense* related species (*M. abscessus, M. bolletii, M. chelonae,* and *M. fortuitum*) was designed by using MapDraw (version 3.14; DNASTAR, Madison, Wis.). To verify the authenticity of the PRA algorithm for Type II separation, it was applied to six Type strains (*M. abscessus* ATCC 19977^T^, *M. bolletii* CIP 108541^T^, *M. chelonae*, *M. fortuitum*, *M. massiliense* CIP 108297^T^) and 65 *M. massiliense* clinical isolates. All of the samples were blind tested. Briefly, ten microliters of 644-bp *hsp65* PCR products, 2 U of *Hinf* I restriction enzyme, and a restriction buffer were transferred into a microcentrifuge tube, and distilled water was added to a final volume of 20 µl. Digestion was performed for 2 h at 37°C in a water bath. After digestion, the mixtures were electrophoresed in 2% agarose gel with 100 bp ladder DNA marker.

### Nucleotide Sequencing

PCR products were purified using Qiaex II gel extraction kits (Qiagen, Hilden, Germany) and then sequenced directly using forward and reverse primers with an Applied Biosystems automated sequencer (model 377) and BigDye Terminator cycle sequencing kits (Perkin-Elmer Applied Biosystems, Warrington, United Kingdom). Both strands were sequenced as a crosscheck.

### Sequence Analysis

Determined partial *rpoB* (711-bp), and *hsp65* (603-bp) sequences were aligned using the ClustalW algorithm in MEGA4 [47]. A phylogenetic tree based on the *rpoB* and *hsp65* gene sequences was constructed by the neighbor-joining [48] and maximum-parsimony [49] methods within the MEGA 4 program [47]. The constructed neighbor-joining tree was evaluated by bootstrap value calculated from 1,000 replicates [13]. Separately, the 23 S rRNA gene sequences of 65 *M. massiliense* clinical isolates were analyzed to observe any point mutation at the adenine at position 2058 (A2058) or at A2059 in the peptidyltransferase region of the 23 S rRNA gene [50].

### Nucleotide Sequence Accession Numbers

Determined *rpoB* and *hsp65* sequences that were different from reference strains were deposited in GenBank under accession no. JQ081974 to JQ082103.

### Statistical Analyses

Results were expressed as percentages. The differences between categorical variables were analyzed using the Chi-square test. For continuous variables the Student’s *t*-test was used when the data showed a normal distribution, or the Mann-Whitney *U* test was used when the data was not normally distributed. A *p*-value of <0.05 (two-tailed) was considered to be statistically significant.

## Supporting Information

Figure S1 Mycolic acid profiles of *M. massiliense* strains. Comparison of mycolic acid profiles of (A) *M. massiliense* CIP 108297^T^, (B) 50375 (Type I), (C) 51843 (Type I), (D) 52444 (Type I), (E) 50594 (Type II), (F) 51048 (Type II), and (G) 52188 (Type II) obtained from HPLC analysis. The relative retention time is indicated for each peak. LMW, Low-molecular-weight standard; HMW, High-molecular-weight standard. The asterisks represent a unique peak in Type II HPLC profiles compared with *M. massiliense* CIP 108297^T^ and Type I strains.
(TIF)

Table S1 The *rpoB* and *hsp65* genotypes, *Hinf* I PRA patterns and colony morphology of 65 *M. massiliense* clinical isolates.
(XLSX)

Table S2 Details of cultural and biochemical characteristics. Cultural and biochemical characteristics of *M. abscessus* ATCC 19977^T^, *M. bolletii* CIP 108541^T^, *M. massiliense* CIP 108297^T^, Type I strains (50375, 51843, 52352, and 52444) and Type II strains (50594, 51048, 52188, and 52265). Details of biochemical and cultural results are shown in text. ++, good growth, +, positive/growth; −, negative/no growth; ±, variable. 1, *M. abscessus* ATCC 19977^T^; 2, *M. bolletii* CIP 108541^T^, 3, *M. massiliense* CIP 108297^T^; 4, 50375 (Type I); 5, 51843 (Type I); 6, 52352 (Type 1); 7, 52444 (Type I); 8, 50594 (Type II); 9, 51048 (Type II); 10, 52188 (Type II); 11, 52265 (Type II).
(DOCX)

Table S3 Details of the antibiotic susceptibility profiles. Comparison of the antibiotic susceptibility test results among *M. abscessus* ATCC 19977^T^, *M. bolletii* CIP 108541^T^, *M. massiliense* CIP 108297^T^, Type I strains (50375, 51843, 52352, and 52444) and Type II strains (50594, 51048, 52188, and 52265). ‡ Ami, Amikacin; Cef, Cefoxitin; Cip, Ciprofloxacin; Cla, Clarithromycin; Dox, Doxycycline; Imi, Imipenem; Mox, Moxifloxacin; Rif, Rifampin; Sul, Sulfamethoxazole; Tob, Tobramycin; Emb, Ethambutol.
(DOCX)
