# Correction: Discovery of a Novel *hsp65* Genotype within *Mycobacterium massiliense* Associated with the Rough Colony Morphology

**DOI:** 10.1371/annotation/e3c063c3-fd5a-4ce0-83d9-9a8891d3d11f

**Published:** 2012-09-05

**Authors:** Byoung-Jun Kim, Su-Yeon Yi, Tae-Sun Shim, Seung Yeon Do, Hee-Kyung Yu, Young-Gil Park, Yoon-Hoh Kook, Bum-Joon Kim

There was an error in the Funding statement. The correct funding statement is: This work was supported by a National Research Foundation of Korea (NRF) grant (No. 2010-0014269), Republic of Korea. The funders had no role in study design, data collection and analysis, decision to publish, or preparation of the manuscript.

