# Supplementary figures and images for: Discovery of a Novel hsp65 Genotype within Mycobacterium massiliense Associated with the Rough Colony Morphology
Source: PLoS One. 2012 Jun 5;7(6):e38420. doi: 10.1371/journal.pone.0038420 (PMC3367924; doi:10.1371/journal.pone.0038420)

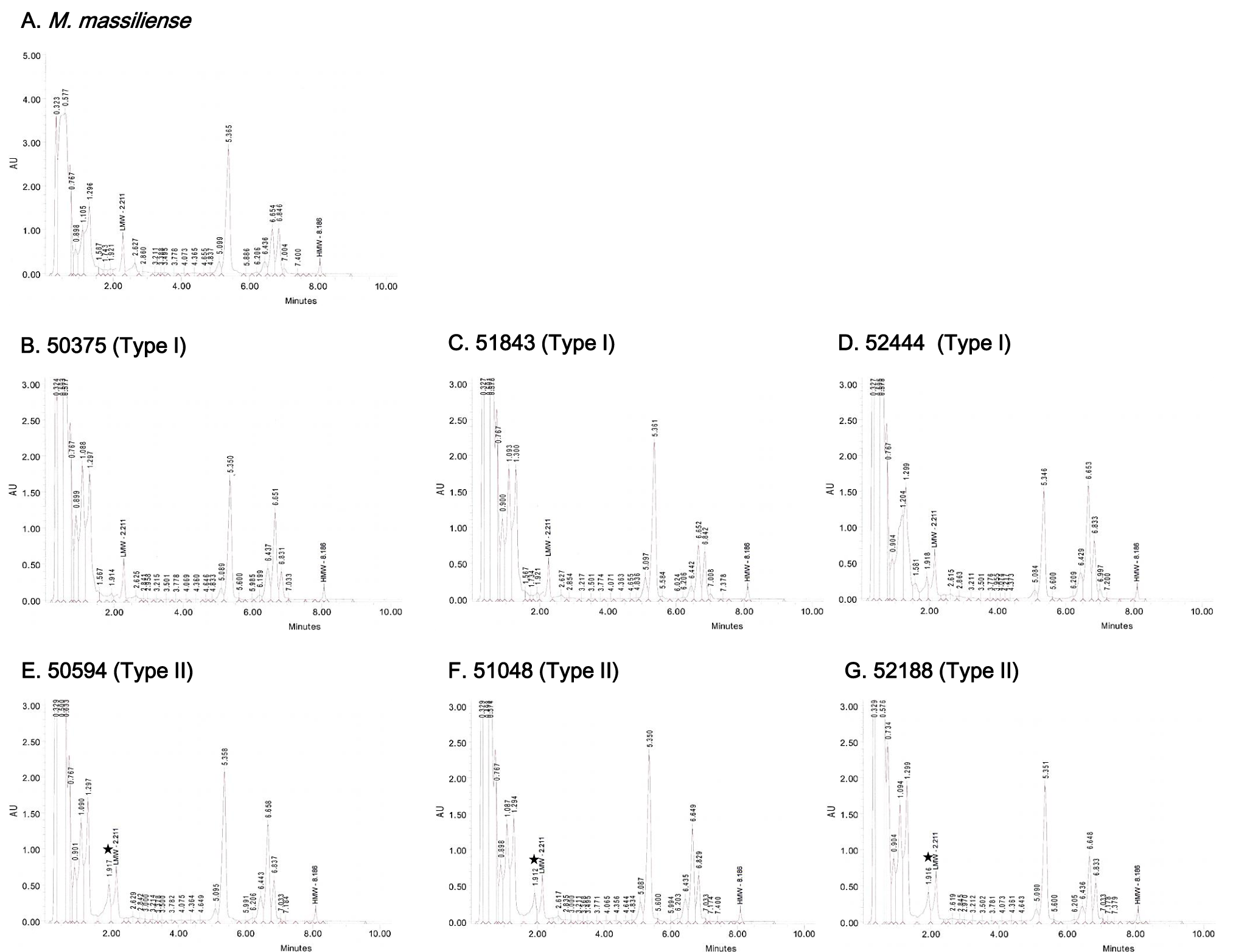

Supplement: Figure S1 — Mycolic acid profiles of M. massiliense strains. Comparison of mycolic acid profiles of (A) M. massiliense CIP 108297T, (B) 50375 (Type I), (C) 51843 (Type I), (D) 52444 (Type I), (E) 50594 (Type II), (F) 51048 (Type II), and (G) 52188 (Type II) obtained from HPLC analysis. The relative retention time is indicated for each peak. LMW, Low-molecular-weight standard; HMW, High-molecular-weight standard. The asterisks represent a unique peak in Type II HPLC profiles compared with M. massiliense CIP 108297T and Type I strains. (TIF) [file pone.0038420.s001.tif]
