# Supplementary material for: Discovery of a Novel hsp65 Genotype within Mycobacterium massiliense Associated with the Rough Colony Morphology
Source: PLoS One. 2012 Jun 5;7(6):e38420. doi: 10.1371/journal.pone.0038420 (PMC3367924; doi:10.1371/journal.pone.0038420)
Supplement: Table S2 — Details of cultural and biochemical characteristics. Cultural and biochemical characteristics of M. abscessus ATCC 19977T, M. bolletii CIP 108541T, M. massiliense CIP 108297T, Type I strains (50375, 51843, 52352, and 52444) and Type II strains (50594, 51048, 52188, and 52265). Details of biochemical and cultural results are shown in text. ++, good growth, +, positive/growth; −, negative/no growth; ±, variable. 1, M. abscessus ATCC 19977T; 2, M. bolletii CIP 108541T, 3, M. massiliense CIP 108297T; 4, 50375 (Type I); 5, 51843 (Type I); 6, 52352 (Type 1); 7, 52444 (Type I); 8, 50594 (Type II); 9, 51048 (Type II); 10, 52188 (Type II); 11, 52265 (Type II). (DOCX) [file pone.0038420.s003.docx]

| Characteristics | 1 | 2 | 3 | 4 | 5 | 6 | 7 | 8 | 9 | 10 | 11 |
| --- | --- | --- | --- | --- | --- | --- | --- | --- | --- | --- | --- |
| Growth at: |  |  |  |  |  |  |  |  |  |  |  |
| 25 ℃ | ++ | ++ | ++ | ++ | +++ | +++ | ++ | ++ | +++ | ++ | ++ |
| 37 ℃ | +++ | +++ | +++ | ++ | ++ | ++ | ++ | ++ | +++ | ++ | ++ |
| 45 ℃ | – | – | – | – | – | – | – | – | – | – | – |
| Growth detectable after: |  |  |  |  |  |  |  |  |  |  |  |
| < 7 days | +++ | +++ | +++ | ++ | ++ | ++ | + | + | ++ | ++ | ++ |
| > 7 days | +++ | +++ | +++ | ++ | ++ | +++ | ++ | ++ | ++ | ++ | ++ |
| Morphology^*^ | SWY | SWY | SWY | SWY | SWY | SWY | IWY | RWY | RWY | RWY | RWY |
| Pigmentation^†^ | N | N | N | N | N | N | N | N | N | N | N |
| Nitrate reductase | – | – | – | – | – | – | – | – | – | – | – |
| Arylsulfatase |  |  |  |  |  |  |  |  |  |  |  |
| 3 days | + | + | + | + | ± | ± | + | + | + | + | + |
| 14 days | + | ++ | +++ | ++ | +++ | ++ | +++ | +++ | ++++ | +++ | +++ |
| Catalase |  |  |  |  |  |  |  |  |  |  |  |
| Heat stable (68℃) | + | – | – | – | ± | + | ± | + | + | + | + |
| Room temperature | + | ++ | ± | ++ | +++ | +++ | ++ | ++ | +++ | + | + |
| Tellurite reductase | ++ | + | + | – | – | – | – | – | ± | – | ± |
| Tween hydrolysis |  |  |  |  |  |  |  |  |  |  |  |
| < 5 days | – | – | – | – | – | – | – | – | – | – | – |
| > 10 days | – | – | – | ± | + | ± | + | + | + | ± | ± |
| Urease | + | + | + | + | ± | + | + | ± | + | + | + |
| Growth with: |  |  |  |  |  |  |  |  |  |  |  |
| 10 mg TCH ml^-1^ | ++ | ++ | ++ | + | ++ | + | + | + | ++ | + | + |
| 500 mg PNB ml^-1^ | + | + | +++ | +++ | + | ++ | + | + | + | + | ++ |
| 5% NaCl | ++ | + | + | + | + | + | – | – | + | + | + |
| Growth on: |  |  |  |  |  |  |  |  |  |  |  |
| MacConkey agar |  |  |  |  |  |  |  |  |  |  |  |
| Picric acid |  |  |  |  |  |  |  |  |  |  |  |
| API ZYM kit |  |  |  |  |  |  |  |  |  |  |  |
| Alkaline phosphatase | – | – | – | – | – | – | + | + | – | – | – |
| Esterase (C4) | + | + | + | + | + | + | + | + | + | + | + |
| Esterase lipase (C8) | + | + | + | + | + | + | + | + | + | + | + |
| Lipase (C14) | – | – | + | – | – | + | – | – | + | + | – |
| Leucine arylamidase | + | + | + | + | + | + | + | + | + | + | + |
| Valine arylamidase | – | – | – | – | – | – | – | – | – | – | – |
| Cystine arylamidase | – | – | + | – | + | – | + | + | – | – | – |

1, *M. abscessus* ATCC 19977^T^; 2, *M. bolletii* CIP 108541^T^, 3, *M. massiliense* CIP 108297^T^; 4, 50375 (Type I); 5, 51843 (Type I); 6, 52352 (Type 1); 7, 52444 (Type I); 8, 50594 (Type II); 9, 51048 (Type II); 10, 52188 (Type II); 11, 52265 (Type II).
