# Supplementary material for: Discovery of a Novel hsp65 Genotype within Mycobacterium massiliense Associated with the Rough Colony Morphology
Source: PLoS One. 2012 Jun 5;7(6):e38420. doi: 10.1371/journal.pone.0038420 (PMC3367924; doi:10.1371/journal.pone.0038420)
Supplement: Table S3 — Details of the antibiotic susceptibility profiles. Comparison of the antibiotic susceptibility test results among M. abscessus ATCC 19977T, M. bolletii CIP 108541T, M. massiliense CIP 108297T, Type I strains (50375, 51843, 52352, and 52444) and Type II strains (50594, 51048, 52188, and 52265). ‡ Ami, Amikacin; Cef, Cefoxitin; Cip, Ciprofloxacin; Cla, Clarithromycin; Dox, Doxycycline; Imi, Imipenem; Mox, Moxifloxacin; Rif, Rifampin; Sul, Sulfamethoxazole; Tob, Tobramycin; Emb, Ethambutol. (DOCX) [file pone.0038420.s004.docx]

| **Strains** | **MIC (μg/ml)** | | | | | | | | | | |  |
| --- | --- | --- | --- | --- | --- | --- | --- | --- | --- | --- | --- | --- |
|  | Ami^‡^ | Cef | Cip | Cla | Dox | Imi | Mox | Rif | Sul | Tob | Emb | |
| *M. abscessus* | 16 | 32 | >16 | 2 | >32 | 16 | 16 | >16 | >128 | 32 | >32 | |
| *M. bolletii* | 16 | 32 | >16 | 2 | >32 | 8 | 16 | >16 | >128 | >32 | >32 | |
| *M. massiliense* | 16 | 32 | >16 | ≤0.5 | 2 | 32 | 16 | >16 | >128 | 32 | >32 | |
| 50375 (Type I) | 16 | 32 | >16 | ≤0.5 | >32 | 16 | >16 | >16 | >128 | 32 | >32 | |
| 51843 (Type I) | 16 | 32 | >16 | ≤0.5 | >32 | 8 | 16 | >16 | >128 | 32 | >32 | |
| 52352 (Type I) | 8 | 16 | 4 | ≤0.5 | >32 | 4 | 8 | >16 | 64 | 64 | >32 | |
| 52444 (Type I) | 32 | 128 | >16 | 4 | >32 | 64 | >16 | >16 | 64 | >32 | >32 | |
| 50594 (Type II) | 64 | 32 | >16 | ≤0.5 | >32 | >64 | 16 | >16 | >128 | >32 | >32 | |
| 51048 (Type II) | 32 | 128 | >16 | ≤0.5 | >32 | >64 | >16 | >16 | >128 | >32 | >32 | |
| 52188 (Type II) | 16 | 16 | 16 | ≤0.5 | >32 | 16 | 8 | >16 | >128 | 16 | >32 | |
| 52265 (Type II) | 16 | 16 | 16 | ≤0.5 | >32 | 8 | 16 | >16 | >128 | >32 | >32 | |

‡ Ami, Amikacin; Cef, Cefoxitin; Cip, Ciprofloxacin; Cla, Clarithromycin; Dox, Doxycycline; Imi, Imipenem; Mox, Moxifloxacin; Rif, Rifampin; Sul, Sulfamethoxazole; Tob, Tobramycin; Emb, Ethambutol.
